# Calpain-Calcineurin-Nuclear Factor Signaling and the Development of Atrial Fibrillation in Patients with Valvular Heart Disease and Diabetes

**DOI:** 10.1155/2016/4639654

**Published:** 2016-03-31

**Authors:** Yong Zhao, Guo-ming Cui, Nan-nan Zhou, Cong Li, Qing Zhang, Hui Sun, Bo Han, Cheng-wei Zou, Li-juan Wang, Xiao-dong Li, Jian-chun Wang

**Affiliations:** ^1^Department of Geriatric Cardiology, Shandong Provincial Hospital Affiliated to Shandong University, Jinan 250021, China; ^2^Shandong Medicinal Imaging Research Institute, Jinan 250021, China; ^3^Intensive Care Unit, Shouguang People's Hospital, Weifang 262700, China; ^4^Department of Cardiology, Jinan Central Hospital Affiliated to Shandong University, Jinan, Shandong 250013, China; ^5^Department of Cardiac Surgery, The 4th Hospital of Jinan, Jinan 250031, China; ^6^Department of Cardiac Surgery, Shandong Provincial Hospital Affiliated to Shandong University, Jinan 250021, China

## Abstract

Calpain, calcineurin (CaN), and nuclear factor of activated T cell (NFAT) play a key role in the development of atrial fibrillation. Patients with valvular heart disease (VHD) are prone to develop atrial fibrillation (AF). Thus, our current study was aimed at investigating whether activation of calpain-CaN-NFAT pathway is associated with the incidence of AF in the patients with VHD and diabetes. The expressions of calpain 2 and alpha- and beta-isoforms of CaN catalytic subunit (CnA) as well as NFAT-c3 and NFAT-c4 were quantified by quantitative reverse transcription-polymerase chain reaction in atrial tissues from 77 hospitalized patients with VHD and diabetes. The relevant protein content was measured by Western blot and calpain 2 in human atrium was localized by immunohistochemistry. We found that the expressions of calpain 2, CnA alpha and CnA beta, and NFAT-c3 but not NFAT-c4 were significantly elevated in the samples from patients with AF compared to those with sinus rhythm (SR). Elevated protein levels of calpain 2 and CnA were observed in patients with AF, and so was the enhanced localization of calpain 2. We thereby concluded that CaN together with its upstream molecule, calpain 2, and its downstream effector, NFAT-c3, might contribute to the development of AF in patients with VHD and diabetes.

## 1. Introduction

Atrial fibrillation (AF) is the most common abnormal heart rhythm in human and imposes a substantial burden on population health. The odds of developing a stroke are almost 5-fold higher in patients with AF than in those free of AF [1]. However, the underlying pathophysiological mechanisms of AF are not entirely understood. It may be associated with ion channel dysfunction, Ca^2+^-handling abnormalities, structural remodeling, and autonomic neural dysregulation [2].

Calpains represent a family of intracellular Ca^2+^-activated cysteine proteases and the 15 family members have been proposed to be actively involved in the occurrence of AF [3]. Calcineurin (CaN) is a Ca^2+^/calmodulin activated serine/threonine protein phosphatase and can be activated by endogenous calpain in human heart [4]. It is composed of a ≈60-kDa catalytic A subunit (CnA) and a 19 kDa regulatory B subunit (CnB). Three isoforms have been identified in CnA (*α*, *β*, and *γ*) and 2 isoforms in CnB (CnB1 and CnB2) [5]. CnA has been reported to modulate transcription through dephosphorylating and facilitating translocation of nuclear factor of activated T cells (NFATs). Previous studies also indicated that CaN activity was increased following NFAT-c3 and NFAT-c4 translocation into the nucleus in a porcine model of AF, suggesting that the activation of CaN-NFAT signal transduction pathway might be involved in the pathogenesis of AF [6].

Many pathological processes contribute to the development of AF. For example, valvular heart disease (VHD) has been identified to be a risk factor of AF [7]. In addition, a prospective cohort study suggested that diabetes also significantly increased the incidence of AF by 35% [8]. Thus, in the current study, we aimed to investigate the relationship between calpain-CaN-NFAT pathway and the development of AF in patients with VHD and diabetes. We evaluated the expressions of calpain, CaN, and NFATs in atrial tissues from patients with and without chronic persistent AF.

## 2. Methods

### 2.1. Patients and Atrial Samples

This human study was conducted in accordance with the principles outlined in Declaration of Helsinki. The research protocol was approved by the Ethics Committee of Provincial Hospital Affiliated to Shandong University. Written informed consents for research use of discarded atrial tissues were provided by all the patients or their guardians. Within 2007–2013, left atrial appendages were dissected from 77 patients with VHD and diabetes during the mitral/aortic valve replacement surgeries. All these patients had their cardiac functions in New York Heart Association (NYHA) II-III function class and none of them ever received class I or III antiarrhythmic drugs. These atrial samples were subsequently frozen in liquid nitrogen and stored at −80°C for a further analysis.

Demographic and clinical data were retrieved by a chart review. Blood pressure (BP) was measured with a mercury sphygmomanometer on the right arm of each patient resting in a supine posture. And the heart rate was documented. Prior to the surgeries, overnight fasting blood samples were collected to measure hemoglobin (Hb), alanine transferase (ALT), creatinine (Cr), low-density lipoprotein cholesterol (LDL-C), fast blood glucose (FBG), international normalized ratio (INR), angiotensin I and angiotensin II (Ang I and Ang II), aldosterone, and brain natriuretic peptide (BNP). Atrial fibrillation was confirmed with electrocardiography. Transthoracic echocardiography through Philips Sonos 5500 Ultrasound System (HP Agilent, SC, USA) was operated by an experienced sonographer to assess the diastolic diameter of each heart chamber, the thickness of interventricular septum (IVS) and left ventricular posterior wall (LVPW), the status of valves, and the left ventricular ejection fraction (LVEF).

### 2.2. Gene Expression Measurement by Real-Time Quantitative Reverse Transcription-Polymerase Chain Reaction (qPCR)

Frozen left atrial appendage was thawed and removed from RNAIater stabilization reagent. About 50 mg of specimens was minced on ice and homogenized in 1 mL TRIzol (TaKaRa Bio, Tokyo, Japan). After addition of 200 *μ*L chloroform, the mixture was incubated at room temperature for 3 min and centrifuged at 12,000 g for 15 min at 4°C. Upper aqueous phase (400 *μ*L) was transferred to a 1.5 mL RNase-free tube and mixed with 1 volume of isopropanol. The homogenate sat on ice for 10 min and was centrifuged at 12,000 g for 10 min at 4°C. Total RNA was precipitated and then washed by 1 mL 75% ethanol. After centrifugation at 7,500 g for 5 min at 4°C and addition of ethanol were repeated twice, the recovered RNA pellet was dissolved in diethylpyrocarbonate- (DEPC-) treated sterile water. Total RNA was quantified spectrophotometrically at 260 nm and its quality was validated by the OD_260_/OD_280_ absorption ratio in the range of 1.8 to 2.0.

RNAs were reverse-transcribed into cDNA using PrimeScript*™* RT Master Mix (TaKaRa Bio, Tokyo, Japan). During the synthesis of cDNA, a mix of 5 20 *μ*g RNA, 2 *μ*L 5 ×g DNA Eraser Buffer, and 1 *μ*L gDNA Eraser was incubated in a final volume of 10 *μ*L with RNase free ddH_2_O at 42°C for 4 min to minimize the contamination of genomic DNA. Then 1 *μ*L PrimeScript RT Enzyme Mix I, 1 *μ*L RT Primer Mix, 4 *μ*L 5x PrimeScript Buffer, and 4 *μ*L RNase free ddH_2_O were added for 15-minute incubation at 37°C, followed by inactivation of reverse transcriptase at 85°C for 5 sec, and stored at 4°C.

Real-time quantitative PCR was performed using a LightCycler® 480 Real-Time PCR System (Roche Diagnostics, GmbH, Penzberg, Germany). Briefly, the reaction mixtures (20 *μ*L) included 2 *μ*L cDNA, 10 *μ*L 2x SYBR® Premix Ex Taq*™* (TaKaRa Bio, Tokyo, Japan), and 0.4 *μ*L 10 *μ*mol/L specific sense and antisense primers listed in Table 1. Cycle conditions were predenaturation at 95°C for 30 sec, then 40 cycles of denaturation at 94°C for 5 sec, annealing at 60°C for 20 sec, and extension at 95°C for 5 sec, followed by a final extension at 60°C for 1 min. The specificity of PCR product was examined by 1.5% agarose gel electrophoresis and melting curve analysis. Each assay was analyzed in triplicate, and the relative expression levels were calculated by the 2^−ΔΔCt^ method using *β*-actin as endogenous control for normalization of cDNA contents.

### 2.3. Protein Quantification by Western Blot

About 50 mg frozen samples from left atrial appendage were grinded in liquid nitrogen and subsequently homogenized in 1.5 mL lysis buffer (50 mM Tris-HCl, 100 mM NaCl, 1% NP-40, 0.1% SDS, 1 mM phenylmethylsulfonyl fluoride, pH 7.4). Homogenates were incubated for 30 min on ice and centrifuged at 15,000 g for 30 min at 4°C. The protein content from supernatants was determined using bicinchoninic acid (BCA) method. After being denatured at 100°C for 10 min, extracted proteins (40 *μ*g per lane) were separated by 8%–10% gradient sodium dodecyl sulfate-polyacrylamide gel electrophoresis (SDS–PAGE) at 100 V for 1.5 h and transferred onto polyvinylidene fluoride (PVEF) membranes (Millipore, Massachusetts, USA) at 4°C and 200 mA. Membranes were blocked in 5% w/v nonfat dry milk TBST buffer (Tris-base 1.21 g/L, NaCl 8.78 g/L, 1 mL Tween-20/L, pH 7.6) at room temperature for 1 h and incubated for overnight at 4°C with primary antibodies: mouse anti-human CaN monoclonal antibody (BD Transduction Laboratories, San Diego, CA, USA; 1 : 250), rabbit anti-human calpain 2 polyclonal antibody (Abgent, San Diego, CA, USA; 1 : 1000), and mouse anti-human *β*-actin monoclonal antibody (Zhongshan Jinqiao Bio-Technology Co. Ltd., Beijing, China; 1 : 1000). In the next day, membranes were washed with TBST buffer 3 times and incubated with horseradish peroxidase-conjugated goat anti-rabbit/mouse secondary antibody (Zhongshan Jinqiao Bio-Technology Co. Ltd., Beijing, China; 1 : 1000) at room temperature for 1 h. After another 3 washes with TBST buffer, signals were detected with enhanced chemiluminescence (ECL) HRP substrate (Millipore, Boston, MA, USA) and quantified by densitometry with Fujifilm Las-4000 Luminescent Image Analyzer (Fuji, Tokyo, Japan).

### 2.4. Immunohistochemical Localization of Calpain 2

Dissected tissues were fixed in 10% paraformaldehyde and made into 20 *μ*m thick paraffin-embedded sections. After being heated for 30 min at 65°C, slides were deparaffinized with turpentine oil, rehydrated with gradient ethanols, rinsed with distilled water 3 times for 5 min each, washed with phosphate buffered saline (PBS) for 5 min, and immersed into preheated retrieval solution (98°C, pH = 6.0) for 15 min. Upon cooling to room temperature, slides were rinsed gently with PBS 3 times for 5 min each and then incubated with 3% H_2_O_2_ at 37°C for 30 min to quench endogenous peroxidase activity. Rinsed for another 3 times with PBS, the slides were added with normal goat serum (Zhongshan Jinqiao Bio-Technology Co. Ltd., Beijing, China) to block nonspecific antigens for 30 min at 37°C. Excess blocking serum was wiped away and the slides were incubated with rabbit polyclonal primary antibodies to calpain 2 (Abgent, San Diego, CA, USA; 1 : 100) overnight at 4°C. After incubation at 37°C for 30 min and 3 washes with PBS, biotinylated secondary antibodies (Zhongshan Jinqiao Bio-Technology Co. Ltd., Beijing, China) were added to incubate at 37°C for 30 min followed by another 3 washes with PBS. The tissue sections were immersed with 3,3′-diaminobenzidine (DAB) Chromogen Solution (Zhongshan Jinqiao Bio-Technology Co. Ltd., Beijing, China) for 3 min and mounted with nuclear counterstain hematoxylin for 3 min. After being dehydrated with ethanol and cleared with turpentine oil, stained tissue was covered with a coverslip and visualized under a light microscope. The specificity of immunostaining was controlled by substituting the primary antibodies with PBS. Calpain 2-positive cells were identified by the presence of brown granules in the cytoplasm. Image-Pro Plus Version 6.0 image analysis system (Media Cybernetics, Inc., Silver Spring, MD, USA) was adopted to evaluate semiquantitatively the average optical density (OD) of the positive stained areas in 3 random visual fields (magnification, ×400) from each histological section.

### 2.5. Statistical Analysis

Continuous variables were expressed as Mean ± Standard Deviation (SD). SPSS 17.0 (SPSS Inc., Chicago, IL, USA) was used for data analysis. The difference between two groups was evaluated by unpaired Student's *t*-test and the comparison among multigroups was tested by one-way analysis of variance (ANOVA). Categorical variables were summarized as frequency and percentage and evaluated using Chi-square or Fisher's exact test. Pearson correlation coefficient (*r*) was used to assess the association between parameters. *P* < 0.05 was considered as significant difference.

## 3. Results

### 3.1. Clinical Characteristics of Patients with AF and SR

The patients include 42 males and 35 females of the age of 50.73 ± 7.65 years. Forty-five of them were diagnosed with AF and 32 with sinus rhythm (SR). Tables 2 and 3 show the preoperative clinical and laboratory characteristics of these patients. The rest heart rates and levels of INR, BNP, and Ang I and Ang II were significantly higher in patients with AF compared to those with SR. These increased parameters were paralleled with exacerbated cardiac dysfunction characterized with augmented left and right atrium and attenuated left ventricular ejection fraction (LVEF).

### 3.2. The Gene Expressions of Calpain 2, CaN, and NFATs

**Figure 1:**
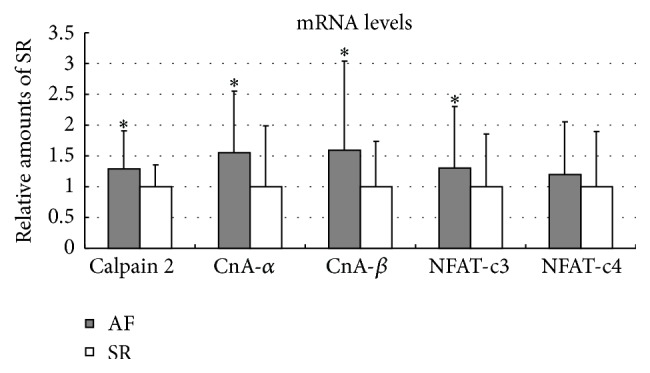
The mRNA expressions of calpain 2, CaN, and NFATs in left atrial tissues isolated from patients with AF and SR. In comparison with SR subjects, significantly increased mRNA levels of calpain 2 (128.37 ± 63.01% versus 100 ± 36.07%, *P* = 0.015), CnA *α* (154.90 ± 100.38% versus 100 ± 98.44%, *P* = 0.020), CnA *β* (159.16 ± 144.34% versus 100 ± 73.36%, *P* = 0.037), and NFAT-c3 (130.79 ± 100.00% versus 100 ± 85.48%, *P* = 0.028), but not NFAT-c4 (119.40 ± 85.51% versus 100 ± 89.82%, *P* = 0.340), were observed in patients with AF. ^*∗*^ *P* < 0.05 versus SR. AF indicates atrial fibrillation; CnA alpha and CnA beta indicate *α* and *β* isoforms of catalytic A subunit in calcineurin; NFAT indicates nuclear factor of activated T cells; SR indicates sinus rhythm.

At the transcription level, the expressions of calpain 2 (128.37 ± 63.01% versus 100 ± 36.07%, *P* = 0.015), CnA *α* (154.90 ± 100.38% versus 100 ± 98.44%, *P* = 0.020), CnA *β* (159.16 ± 144.34% versus 100 ± 73.36%, *P* = 0.037), and NFAT-c3 (130.79 ± 100.00% versus 100 ± 85.48%, *P* = 0.028) were significantly increased in patients with AF compared to those with SR. However, there was no statistical difference in the expression of NFAT-c4 between these two groups (119.40 ± 85.51% versus 100 ± 89.82%, *P* = 0.340; Figure 1). These findings suggested that Calpain 2, CnA *β*, and NFAT-c3 rather than NFAT-c4 might contribute toward the development of AF.

### 3.3. The Protein Expressions of Calpain 2 and CaN

**Figure 2:**
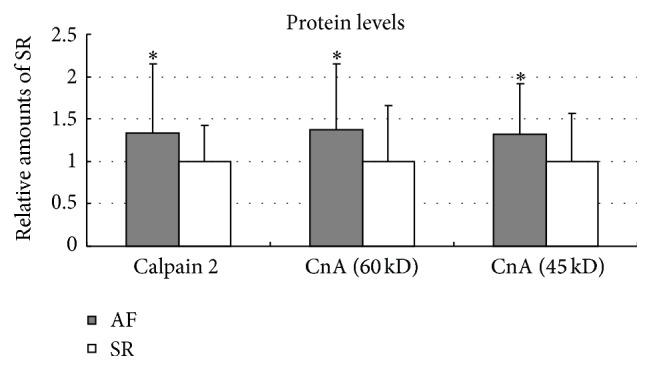
The protein expressions of calpain 2, full-length CnA (60 kD), and truncated CnA (45 kD) in left atriums of patients with AF and SR. In the protein levels, statistical elevation of calpain 2 (133.68 ± 80.82% versus 100 ± 42.12%, *P* = 0.020), full-length CnA (60 kD) (137.47 ± 77.61% versus 100 ± 65.17%, *P* = 0.029), and truncated CnA without autoinhibitory domain (45 kD) (132.40 ± 59.94% versus 100 ± 56.19%, *P* = 0.019) were found in patients with AF in comparison with SR subjects. ^*∗*^ *P* < 0.05 versus SR. AF indicates atrial fibrillation; CnA indicates catalytic A subunit of calcineurin; SR indicates sinus rhythm.

As demonstrated in Figure 2, the protein levels of calpain 2 in atrial samples from patients with AF were higher than those with SR (133.68 ± 80.82% versus 100 ± 42.12%, *P* = 0.020). In addition, the protein expression increased in both full-length CnA (60 kD) and its truncated fragment without autoinhibitory domain (45 kD) in patients with AF (137.47 ± 77.61% versus 100 ± 65.17%, *P* = 0.029; 132.40 ± 59.94% versus 100 ± 56.19%, *P* = 0.019, resp.). Such results further indicated that calpain 2-CaN pathway might regulate the development of AF through its transcriptional effects.

### 3.4. Localization of Calpain 2 in Left Atrium

**Figure 3:**
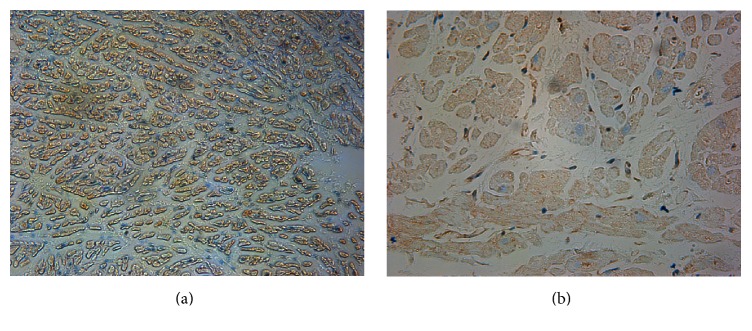
Immunohistochemical localization of calpain 2 in atrial cardiomyocytes ((a) AF group, ×200; (b) SR group, ×400). Calpain 2, brown-stained granules, was localized in the cytoplasm of atrial myocytes, but not in endocardium or epicardium. Enhanced expression of calpain 2 was observed in samples from patients with AF compared to those from SR group. AF indicates atrial fibrillation; SR indicates sinus rhythm.

Although cardiomyocyte is most dominant in the heart, other cell types exit, such as smooth muscle cells, endothelial cells, and fibroblasts. Thus, in our current study, we investigated the localization of calpain 2 by immunohistochemistry staining and defined whether it is involved in the development of AF in cardiomyocytes. As shown in Figure 3, brown granules indicating calpain 2 were only visualized in the cytoplasm of atrial myocytes, but not in endocardium or epicardium. An increase in calpain 2 staining was also observed in cardiac samples from patients with AF compared to those from SR group (Figure 3).

## 4. Discussion

Atrial fibrillation (AF) is a common severe arrhythmia and often develops in patients with VHD and diabetes. Permanent AF is associated with decreased cardiac function and is an independent predictor of heart failure [9]. Calpains have been proposed to be actively involved in the occurrence of AF; however, the molecular mechanisms are not well clarified in human beings. Thus, our current study investigated the role of calpain 2-CaN-NFAT pathway in mediating the development of AF in the patients with VHD and diabetes. Our results indicated that the gene expressions of calpain 2, CaN, and NFAT-c3 but not NFAT-c4 were significantly increased in the left atrial tissue of AF patients with VHD and diabetes compared to those with SR. The upregulated gene expressions of calpain 2 and CaN responded to an augmented protein levels, and the increased calpain 2 was localized in atrial cardiomyocytes from AF samples, which suggested a positive association of cardiac calpain-CaN-NFAT signaling with AF.

Calpains 1 (or calpain *μ*) and 2 (or calpain m) are the two major isoforms of calpain and ubiquitously expressed in the heart. However, calpain 1 was usually activated with a micromolar level of Ca^2+^, while the activation of calpain 2 requires a higher or pathological millimolar level of intracellular Ca^2+^ [10, 11]. Goette et al. previously reported that the level of calpain 1 was increased more than 3 times and the calpain enzymatic activity was doubled in the atrial tissue samples from patients with AF compared to those in SR group [12]. However, the mRNA expressions of calpains 1 and 2 and calpain 2 protein levels between AF and SR patients are identical. A following study further proved the effects of calpain 1 in AF [13]. They found that upregulation of calpain 1, rather than calpain 2, contributes to the activation of calpain. However, our current study indicated that AF is associated with upregulated gene and protein expressions of calpain 2 in the heart tissue. The different results from our current study and other previous reports are probably associated with different cardiac functions and plasma Ang II levels in recruited patients. In our recruited patients, NYHA class III is more prevalent and the patients with AF have a lower LVEF compared to those with SR. Indeed, calpain 2 was significantly increased in NYHA class III or IV failing hearts, but not in class II or nonfailing ones [14]. Furthermore, our studies showed that the levels of Ang I and Ang II were significantly elevated in AF group. A previous finding suggested that Ang II regulates calpains in ventricular remodeling [14].

Calpains 1 and 2 can cause limited proteolysis of full-length CaN in a Ca^2+^-dependent manner without the presence of calmodulin [4]. AF induces intracellular calcium overload in atrial cardiomyocytes [15] which may facilitate the activation of CaN-NFAT pathway. The right atrial tissues isolated from VHD patients with AF had upregulated gene and protein expressions in CaN catalytic and regulatory subunits which contribute to increased activity of the catalytic subunit [16]. NFAT-c3 and NFAT-c4 are the downstream effectors of CaN, which accumulate in the nuclei during AF due to the increased enzymatic activity of CaN [6]. CnA phosphatase activity and CnA *β*-isoform protein contents were enhanced in patients with chronic AF [17], and the nuclear accumulation of NFAT-c3 protein and the mRNA expression of NFAT-c3 were also upregulated [17]. Our present study demonstrated that the gene and protein expressions of CnA and NFAT-c3 were dramatically elevated in AF patients with VHD and diabetes compared to those with SR. However, it is unclear whether CnB is also upregulated in AF, though CnA and CnB are coexpressed and mutually stabilize each other [18].

It should be noted that pharmacological therapy prior to cardiac surgery might affect calpain pathway in our study. Currently, we do not know whether *β*-blockers or diuretics regulate calpain expression; however, digoxin and calcium channel blockers have been tested not affecting calpain 1 expression [12]. Moreover, our recruited patients did not include those with cardiac function NYHA class I or IV. Our future study will expand the patient cohort and identify the role of calpain-CaN-NFAT signaling in mediating AF in all the NYHA classes.

## Figures and Tables

**Table 1:** Primer sequences were used in qPCR experiments.

Gene	Product size (bp)	Primer sequence
Calpain 2	165	F: 5′-CTGGGGCTGAAGGAGTTCTAC
		R: 5′-GATGACTTGGTGGAGTTGACAG

CnA *α* isoform	112	F: 5′-CAGGAACATTTCACTCACAACACAG
		R: 5′-CGTGGGCTCGGAGTATAGATAACA

CnA *β* isoform	119	F: 5′-TGGATGTCTTCACGTGGTCTTTAC
		R: 5′-ATCAAACTGGTCTTCACCTTCAGTC

NFAT-c3	137	F: 5′-CATCGAGCCCATTATGAAACTGAA
		R: 5′-CGATCATCTGCTGTCCCAATAAAC

NFAT-c4	97	F: 5′-AGCCTGACACACCGTAGGTACTGA
		R: 5′-AGTGCAAATGCCCGGAATG

*β*-actin	366	F: 5′-ACACTGTGCCCATCTACGAGGGG
		R: 5′-ATGAGTGAGTTGAAGGTAGTTTCGTGGAT

CnA, subunit A of calcineurin; F, forward primer; NFAT, nuclear factor of activated T cells; R, reverse primer.

**Table 2:** Clinical assessments of patients.

	AF (*n* = 45)	SR (*n* = 32)	*P*
Age (years)	51.20 ± 6.33	50.06 ± 9.26	0.524
Sex (male, %)	24 (53.33%)	18 (56.25%)	0.800
Cigarette smoking	9 (20.00%)	7 (21.88%)	0.842
Alcohol drinking	13 (28.89%)	11 (34.38%)	0.609
Diseases			
MS	14 (31.11%)	10 (31.25%)	0.505
MR	22 (48.89%)	18 (56.25%)	
MS + MR	6 (13.33%)	4 (12.50%)	
MS + MR + AS/AR	3 (6.67%)	0	
Physical examination			
Systolic BP (mmHg)	120.22 ± 8.40	123.31 ± 12.62	0.201
Diastolic BP (mmHg)	75.98 ± 8.01	73.06 ± 10.54	0.172
Rest heart rates (bpm)	81.13 ± 14.74	74.03 ± 8.91	**0.011**
Cardiac function			
NYHA class II	11 (24.44%)	17 (53.13%)	**0.019**
NYHA class III	34 (75.56%)	15 (46.87%)	
Medications			
ACEI (%)	8 (19.03%)	3 (9.38%)	0.345
*β*-blockers (%)	18 (40.00%)	4 (12.50%)	**0.011**
Digoxin (%)	25 (55.56%)	0	**<0.001**
Diuretics (%)	45 (100%)	18 (58.25%)	**<0.001**

ACEI, angiotensin-converting enzyme inhibitors; AF, atrial fibrillation; AR, aortic regurgitation; AS, aortic stenosis; BP, blood pressure; bpm, beats per minute; MR, mitral regurgitation; MS, mitral stenosis; NYHA, New York Heart Association; SR, sinus rhythm.

**Table 3:** Laboratory assessments of patients.

	AF (*n* = 45)	SR (*n* = 32)	*P*
Blood measurements			
Hb (g/L)	129.17 ± 11.84	131.61 ± 8.70	0.353
ALT (U/L)	27.24 ± 15.22	22.86 ± 8.41	0.127
Cr (*μ*mol/L)	87.37 ± 13.51	82.04 ± 19.64	0.182
LDL-C (mmol/L)	2.47 ± 0.69	2.45 ± 0.57	0.887
FBG (mmol/L)	8.81 ± 1.28	8.52 ± 0.84	0.257
INR	1.44 ± 0.73	0.99 ± 0.10	**<0.001**
BNP (pg/mL)	139.07 ± 94.06	61.86 ± 39.15	**<0.001**
Ang I (ng/mL)	2.13 ± 0.73	1.65 ± 0.54	**0.003**
Ang II (pg/mL)	328.45 ± 145.16	217.46 ± 138.96	**0.002**
Aldosterone (ng/mL)	0.17 ± 0.03	0.17 ± 0.06	0.840
Echocardiography			
LAD (cm)	5.61 ± 0.88	4.48 ± 1.21	**<0.001**
RAD (cm)	4.88 ± 0.46	4.47 ± 0.64	**0.006**
LVD (cm)	5.15 ± 0.82	5.17 ± 0.91	0.930
RVD (cm)	2.35 ± 0.46	2.32 ± 0.60	0.863
LVPW (cm)	0.85 ± 0.16	0.81 ± 0.20	0.382
IVS (cm)	0.85 ± 0.16	0.84 ± 0.19	0.840
LVEF (%)	51.08 ± 4.62	58.86 ± 4.16	**<0.001**

AF, atrial fibrillation; ALT, alanine transferase; Ang, angiotensin; BNP, brain natriuretic peptide; Cr, creatinine; FBG, fast blood glucose; Hb, hemoglobin; INR, international normalized ratio; IVS, interventricular septum; LDL-C, low-density lipoprotein cholesterol; LAD, left atrial dimension; LVD, left ventricular dimension; LVEF, left ventricular ejection fraction; LVPW, left ventricular posterior wall; RAD, right atrial dimension; RVD, right ventricular dimension; SR, sinus rhythm.
